# Application of Health-Care Networking in COVID-19: A Brief Report

**DOI:** 10.1017/dmp.2020.379

**Published:** 2020-10-12

**Authors:** Punidha Kaliaperumal, Tamorish Kole, Neha Chugh

**Affiliations:** International Committee of Red Cross (ICRC), Geneva, Switzerland; Emergency & Disaster Preparedness, Medeor Hospital, Delhi NCR, India; Medeor Institute of Emergency Medicine, Medeor Hospital, Delhi NCR, India; Quality Department, Medeor Hospital, Delhi NCR, India

**Keywords:** COVID-19, disaster preparedness, health-care networking, pandemic, surge capacity

## Abstract

Health-care systems all over the world are stretched out and being reconfigured to deal with the coronavirus disease 2019 (COVID-19) pandemic. Some countries have flattened the curve, some are still fighting to survive it, and others are embracing the second wave. Globally, there is an urgent need to increase the resilience, capacity, and capability of health-care systems to deal with the current crisis and improve upon the future responses. The epidemiological burden of COVID-19 has led to rapid exhaustion of local response resources and massive disruption to the delivery of care in many countries. Health-care networking and liaison are essential component in disaster management and public health emergencies. It aims to provide logistical support between hospitals; financial support through local or regional governmental and nongovernmental agencies for response; manpower and mechanism for coordination and to implement policies, procedures, and technologies in the event of such crisis.

This brief report describes how 4 independent private hospitals in northern India had adopted the principles of health-care networking, pooled their resources, and scaled up 1 of the partner hospitals as Dedicated COVID-19 Hospital (DCH) to treat moderate to severe category of COVID-19 patients. It brings out the importance of a unique coalition between private and public health-care system.

Health-care systems all over the world are stretched out and being reconfigured to deal with coronavirus disease 2019 (COVID-19) pandemic. Some countries have flattened the curve, some are still fighting to survive it, and others are embracing the second wave. Globally, there is an urgent need to increase the resilience, capacity, and capability of health-care systems to deal with the current crisis and improve upon the future responses. Regional outbreaks, such as the ones in Italy and New York, have shown us the need to anticipate both what the ultimate capacity to provide care will be and when that capacity will be exceeded.^1^

The epidemiological burden of COVID-19 has led to rapid exhaustion of local response resources and massive disruption to the delivery of care in many countries. Hospitals in urban Italy have been challenged by sudden surge of patients similar to Level 5 and 6 mass casualty incidents (MCIs) and requiring state-wide resources aid as per definition (> 1000 patients).^2^

Health-care networking and liaison are essential component in disaster management and public health emergencies, especially in natural disasters, mass casualties, large scale industrial accidents and disease outbreaks. In general, aims of networking are to provide logistical support between hospitals, provide financial support through local or regional governmental and nongovernmental agencies for response, to provide a manpower and mechanism for coordination and to implement policies, procedures, and technologies in the event of such crisis.^3^

One mathematical modeling concluded that an uninterrupted epidemic in India would have resulted in more than 364 million cases and 1.56 million deaths with peak by mid-July of 2020. Scarcity of health-care resources in India would have been a major challenge as the Indian Council of Medical Research (ICMR) estimated that India has approximately 70,000 ICU beds and even fewer ventilators. While the elderly (>60 years) comprise around 10% of the population in India, in the model, they accounted for approximately 43% of all hospital admissions and approximately 82% each of intensive care unit (ICU) admissions and deaths. Therefore, it is evident that that both hospital capacity and capability needed to scale up to meet the challenge.^4^

This brief report describes how 4 independent private hospitals in northern India had adopted the principles of health-care networking, pooled their resources, and scaled up 1 of the partner hospitals as a Dedicated COVID-19 Hospital (DCH) to treat moderate to severe category of COVID-19 patients during this crisis. The Chief Medical Officer (CMO) and other authorities of the concerned district provided the necessary oversight and support during this period (May to July 2020).

## BACKGROUND

In response to the COVID-19 pandemic, China came up with a novel public health concept called Fangcang shelter hospitals (FSHs) in February 2020. FSHs are large-scale temporary hospitals, rapidly built in existing public venues, such as stadiums and exhibition centers. They served to isolate mild to moderate COVID-19 patients to provide medical care, disease monitoring, food, shelter, and social activities.^5^ This model was replicated later in many countries, including India.

In contrast, the designation of large District General Hospitals (DGHs) as “COVID-Hospitals” model was part of a framework developed in Bergamo, Italy by national authorities to manage the recent crisis of COVID-19. All hospitals spaces, specially the operating rooms were converted into additional intensive care units (ICUs). Likewise, hospital staff including the surgical teams were re-allocated to run the clinical operations.^2^

The Emergency Medical Relief (EMR) division of the Ministry of Health & Family Welfare (MOHFW), Directorate General of Health Services, Government of India issued a Guidance document on appropriate management of suspected/confirmed cases of COVID-19 in April 2020 and assigned that for severe COVID-19 cases; DCHs can be set up using the entire hospital or a separate block in a hospital. These facilities were designated as referral centers for COVID Care Centers and Dedicated COVID Health Care Centers, which were treating mild and moderate patients.^6^

DCH was defined as health-care facilities (hospitals – government or private) that would offer comprehensive care primarily for severe COVID-19 cases, had dedicated either the full hospital or a separate block with preferably separate entry/exit, would have separate areas for suspected and confirmed cases, would have fully equipped ICUs, ventilators and beds with ensured oxygen support, and follow strict infection control practices.

Per the MOHFW, a severe case was defined as a patient who is clinically demonstrating the signs of severe pneumonia with respiratory rate ≥30/min and/or SpO2 < 90% in room air or acute respiratory distress syndrome (ARDS) or septic shock. Such cases would be directly admitted to a DCH’s ICU until such time as test results are obtained. If test results are positive, such patients will remain in COVID-19 ICU and receive treatment per standard treatment protocol. Patients testing negative will be managed with adequate infection prevention and control practices.^6^

India was 1 of the countries to initiate a stringent nationwide lockdown early in the phase of the pandemic. This helped in not only delaying the peak but also bought time for the health-care system to prepare and brace itself for the eventuality. Anticipating the surge in the National Capital Region (NCR) of India, the state and central government started looking into possibility of setting up DCH in the region. While the government was looking at converting the state-run hospitals, 4 private hospitals in Gurgaon (part of NCR) came up with the novel concept of setting up a DCH in 1 of the partner hospitals (who’s promoter decided to dedicate the facility for COVID-19 treatment) to quickly scale up the facility to be able to treat severe COVID-19 patients in the region. Similar to the Multi Hospital Network (MHN) in Bergamo, Italy^2^; coordinated response between the hospitals was planned by pooling manpower and resources, strengthening procurement strategies, framing patients transfer protocols between the centers, allocation of specialized services, and optimization of intensive care expertise.

This proposal was readily accepted by the local administration, which facilitated the infrastructure modifications with prompt approvals and go-aheads during the phase of complete lockdown. The project was further helped by the “Gurgaon COVID-19 Volunteers Group,” a group formed by few Chief Executive Officers (CEOs) from leading corporations within the city and an NGO “I am Gurgaon” to initiate a large fund-raising campaign for the hospital and treatment of pateints.^7^

## IMPLEMENTATION

A task force was constituted involving experts (Clinicians, Intensivists, Infection Control Officers, Nursing Leaders, Facility management experts, Biomedical experts etc) from all 4 hospitals. To create an action plan, the 4S Surge System was used which consists of staff (personnel), stuff (supplies and equipment), structure (physical space reallocation), and system level integration (protocols, standard operating procedures [SOPs]).^8^

## STRUCTURE

The hospital campus had 2 buildings: hospital building and annex. All nonmedical and administrative staff were shifted out to the ground floor of annex, which was repurposed into a large co-working space.

As per requirement of DCH, all out-patient services and elective surgeries were directed to partner hospitals or other branches of the designated hospital. Admitted patients were either discharged home or shifted to other branches. In contrast, at Northwell Health, the largest health-care provider in New York, hospitals were using lobbies, conference rooms, and cafeterias to deal with crisis and were handling both COVID and non-COVID patients.^9^

Separate entries were designated for staff, patients, and biomedical waste. All patients were routed through the emergency department (ED), where a barricaded counter for admission and billing was constructed to minimize transmission to admission desk staff.

A unidirectional flow of staff and patients was ensured, with donning, doffing, and shower areas made in each floor. Elevators were marked clean and contaminated accordingly.

Some construction was done, some out-patient rooms were barricaded with ceiling-to-floor partitions, some patient rooms were used up to make proper doffing areas, opening into anterooms for discarding N95 masks, further opening into shower rooms and subsequent exits on each floor.

Air handling units (AHUs) and air conditionings were modified by shutting off a few units and blocking a few ducts to prevent mixing of air of clean and contaminated zones. Rooms, where this was not possible, were locked, eg, doctor’s room adjacent to the ICU, which shared the same AHU, was locked and the doctor’s duty room was shifted to the clean zone.

Two rooms adjacent to the ICU were made negative pressure for performing aerosol generating procedures (AGPs). Additional HEPA filters were installed to ICU vents. A separate telemedicine unit was set up just outside the ICU in the clean zone for continuous monitoring of patients.

One of the operating rooms (ORs) was made negative pressure to cater to any surgical emergencies in COVID-19 patients. A separate exit of the OR was made functional for staff to directly access the doffing area. One of the labor rooms was made negative pressure to perform deliveries in COVID-19 patients. This was done to minimize aerosol generation.

Barricaded entries with windows for exchange of clean items were constructed between clean and contaminated zones at the entrance of wards and ICU. Contaminated items, such as patient samples and equipment for sterilization were double packaged at the source and exchanged through these windows by placing them on clean baskets covered with yellow bags. The person collecting them would seal the bags on receiving and transport them to the desired location, such as labs or the central sterile supply department (CSSD), where personnel using full PPE would handle them.

The preventive health-care lounge was repurposed into a temporary staff dining area in between shifts as it had a separate entrance and a separate AHU which was shut down.

Heavy exhaust systems were installed in laboratory rooms performing centrifuge and autoclave and in CSSD where contaminated instruments were being washed, to make them negative pressure. A separate laundry disinfection, washing, and drying unit was installed in-house to prevent transmission in a common out-sourced laundry system. The sewage treatment plant (STP) was assessed and upgraded to handle the surge of COVID-19 patients.

During all these constructions and reallocations, fire exit plans were considered and maintained. Entry to the hospital premises was heavily guarded to prevent in-hospital transmission. Only on-duty staffs and COVID-19 positive patients were allowed in. A single attendant was allowed at the time of admission for billing. There was a system of video calling the attendants daily to brief them about their patients’ condition.

As the hospital was being converted to a DCH, the main ED was converted into a COVID-19 ED and the observation area of the ED, with a separate AHU was converted into a general ED to cater to any life-threatening emergencies coming to the hospital in the initial days of the project. After stabilizing, these cases were shifted to partner hospitals.

The hospital started admitting COVID-19 patients on May 11, 2020, with 50 functional beds (12 ICU and 38 ward), which was later upgraded to 120 beds (24 ICU and 96 ward), within 4 weeks of operation, to handle the patient surge.

Patients were referred directly from the office of Chief Medical Officer (CMO) per the guidelines.

## STAFF

All 4 partner hospitals had pooled in their staff and made a composite roster to work in the designated DCH. Initially, according to the ICMR (Indian Council of Medical Research) guidelines, all staffs worked in shifts for 14 days and were quarantined for 14 days to prevent transmission to family members. Later, as the guidelines changed, staff roster was reformatted to optimize the manpower.

All staffs including doctors, nurses, ward helps, housekeeping, etc., had undergone extensive in-time training for adequate handling of COVID-19 patients, PPE donning and doffing, efficient biomedical waste handling, and the changes in the structure and systems.

A hotel across the road from the hospital was linked up to provide accommodation for all on duty staff. A separate hotel farther from the hospital was also linked up to provide accommodation for the quarantined staff. Later, per ICMR, the mandatory quarantine for staff was struck off.

Policies were put in place for any staff showing symptoms, their testing, quarantine, and treatment. Staff Clinic was operational 24 × 7 at the ED, and a helpline number was provided to all staff for self-reporting of symptoms. All staff were treated in the same hospital whenever required.

Associations with other agencies providing nurses, ward helps, and housekeeping staff were made. The new appointees were extensively trained and posted along with old staff to gain familiarity and avoid breaches.

A buddy system of nurses was started. Two nurses of a shift, working at the same station were paired as buddies to keep a check on any breach in each other’s PPE or development of symptoms to report to their line managers for prompt action and isolation. Psychological counseling for all staff was done before the start of this project to address their apprehensions, and the service was made available on a need to need basis from there on.

## STUFF

The structural changes that had to be done needed a lot of material to be procured and engagement of manpower, such as carpenters, which due to the lockdown had run into major roadblocks. Interstate borders were closed, which led to further problems in sticking with timelines. These challenges were sorted out by the involvement of the District Health Authorities in getting interstate passes, liaising with government/public officials, and giving prompt approvals for the project. Engineers were pooled in from partner hospitals for additional support and supervision.

Vendors were sorted for additional items, such as PPEs, N95 masks, face shields, and disposable cutlery for patients, in large quantities. This was again supported by the government authorities as there was a general shortage of PPEs. Logistics were worked out for appointing additional staffs and procuring their uniforms and other necessities.

A list of dedicated medical equipment was prepared and moved to the ICU. These included: video-laryngoscope; blood gas machine; portable X-ray, ultrasound machines; video cameras; and tablets for monitoring and tele-consults.

## SYSTEM

A compendium of policies and protocols was framed using the pre-existing ones of the partner hospitals. However, local changes were incorporated at the departmental level with approval of the taskforce. Emphasis was placed on minimizing exposure of health-care professionals (HCPs) and support staff to infected individuals and at the same time providing efficient and standard treatment to the patients.

The unidirectional flow and demarcation of contaminated and clean zones had put a lot of restrictions on the movement of the staff. Contaminated items, such as patients’ samples, were placed in yellow bags at the source and then double packaged at the barricaded windows between these zones. Minute details were taken into account, such as packaging of file work of discharged or deceased patients in sealed plastic folders to be stored appropriately for a specific amount of time.

Lockers were placed for staff at the entrance of the hospital building for personal belongings. A central system of issue of PPEs and N95 masks for the staff was implemented. Initially, a disinfection process of N95 masks and goggles was being done for reuse. Later, with the change of guidelines, each staff was issued 5 N95 masks for a span of 20 days and to be reused after 4 days of air-drying. Observers were placed both in donning and doffing areas to assist the staff for efficient process.

**FIGURE 1:**
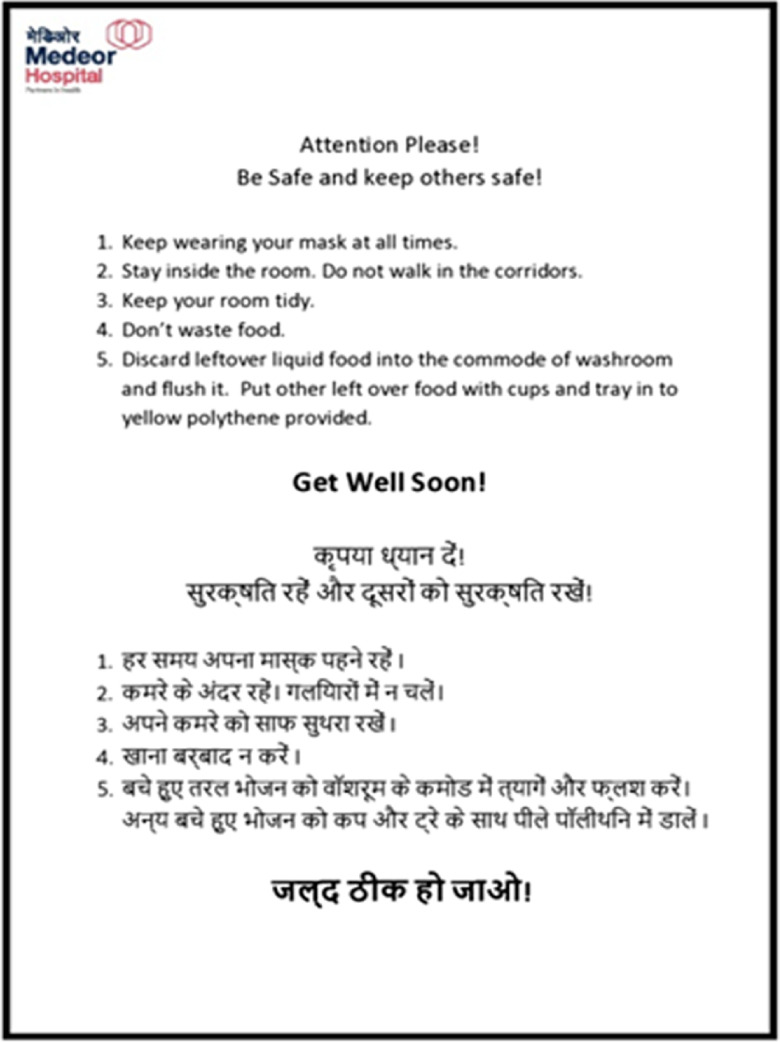
Placard Placed in Patient Room.

**FIGURE 2:**
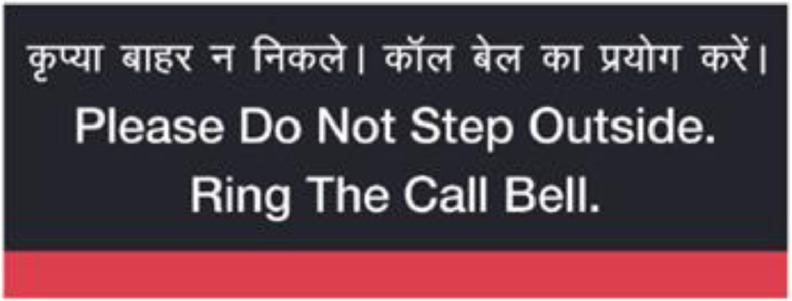
Placard Placed in Patient Room.

Patients’ meals were being served in disposable cutlery to minimize transmission. Placards with instructions on how to dispose of used face tissues, remaining food and cutlery were placed in every patient room. Placards coaxing patients to stay in their own rooms and instructions on how to approach HCPs in case of need was also placed. Samples of placards in Figures 1 and 2.

Policies were put in place for any staff showing symptoms, their testing, quarantine, and treatment. Psychological counselling was made available for staffs on an as needed basis.

## DISCUSSION

Many countries have reconsidered their public health priorities to quickly develop surge capacity and capability to handle this pandemic. The Italian model demonstrated the use of District General Hospitals as DCH to relieve the burden of the pandemic across the National Health System. In contrast, the MOHFW, India model relied on flexibility and allowed creation of DCH in both private and public systems.^6^

Our model was an appropriate and timely intervention using private partnerships, NGO support, personal donors, who came all together to make it successful. Health-care networking is an important strategy to respond to disasters and this model showed the strength and speed of unconventional coalitions that had emerged amidst the COVID-19 pandemic and national lockdown in India.

Also, we encountered anxiety, stress, and fear among the frontline staff; ward helps being most concerned. Regular briefing, addressing their physical and mental well-being, provision of 24 × 7 medical consult at staff clinic, and free testing and treatment helped in many ways to keep them motivated and healthy.

## CONCLUSIONS

Health-care networking and unconventional coalitions can play a synergistic role in pandemic response alongside of national health framework. This is particularly true for countries where private health-care delivers a significant proportion of health care. Governments (local and state) can act as a facilitator to encourage creation of such networks within the existing hospitals.
